# Timing of oxytocin administration to prevent post-partum hemorrhage in women delivered by cesarean section: A systematic review and metanalysis

**DOI:** 10.1371/journal.pone.0252491

**Published:** 2021-06-03

**Authors:** Maria Regina Torloni, Monica Siaulys, Rachel Riera, Ana Luiza Cabrera Martimbianco, Rafael Leite Pacheco, Carolina de Oliveira Cruz Latorraca, Mariana Widmer, Ana Pilar Betrán

**Affiliations:** 1 Department of Obstetrics, Hospital e Maternidade Santa Joana, São Paulo, SP, Brazil; 2 Evidence Based Healthcare Postgraduation Program, Department of Medicine, Universidade Federal de São Paulo, São Paulo, SP, Brazil; 3 Department of Anesthesiology, Hospital e Maternidade Santa Joana, São Paulo, SP, Brazil; 4 Center of Health Technology Assessment, Hospital Sirio-Libanês, São Paulo, SP, Brazil; 5 Universidade Metropolitana de Santos (UNIMES), Santos, SP, Brazil; 6 Centro Universitário São Camilo, São Paulo, SP, Brazil; 7 Department of Reproductive Health and Research, World Health Organization, UNDP/UNFPA/UNICEF/WHO/World Bank Special Programme of Research, Development and Research Training in Human Reproduction (HRP), Geneva, Switzerland; Universita degli Studi di Milano-Bicocca Scuola di Medicina e Chirurgia, ITALY

## Abstract

**Background:**

There is no consensus on the best timing for prophylactic oxytocin administration during cesarean section (CS) to prevent post-partum hemorrhage (PPH).

**Objectives:**

Assess the effects of administrating prophylactic oxytocin at different times during CS.

**Methods:**

We searched nine databases to identify relevant randomized controlled trials (RCT). We pooled results and calculated average risk ratios (RR), mean differences (MD), and 95% confidence intervals (CI). We used GRADE to assess the overall evidence certainty.

**Results:**

We screened 13,389 references and included four trials. We found no statistically significant differences between oxytocin given before versus after fetal delivery on PPH (RR 0.60, 95%CI 0.15–2.47; 1 RCT, N = 300) or nausea/vomiting (RR 1.21, 95%CI 0.69–2.13; 1 RCT, N = 300). There was a significant reduction in the need for additional uterotonics when oxytocin was given immediately before uterine incision versus after fetal delivery (RR 0.37, 95%CI 0.18–0.73; I^2^ = 0%; 2 RCTs; N = 301). Oxytocin given before fetal delivery significantly reduced intra-operative blood loss (MD -146.77mL, 95%CI -168.10 to -125.43; I^2^ = 0%; 3 RCTs, N = 601) but did not change the incidence of blood transfusion (RR 0.50, 95%CI 0.13–1.95; I^2^ = 0%; 2 RCTs, N = 301) or hysterectomy (RR 3.00; 95%CI 0.12–72.77; I^2^ = 0%; 2 RCTs, N = 301). One trial (N = 100) compared prophylactic oxytocin before versus after placental separation and found no significant differences on PPH, additional uterotonics, or nausea/vomiting.

**Conclusions:**

In women having pre-labor CS, there is limited evidence indicating no significant differences between prophylactic oxytocin given before versus after fetal delivery on PPH, nausea/vomiting, blood transfusion, or hysterectomy. Earlier oxytocin administration may reduce the volume of blood loss and need for additional uterotonics. There is very limited evidence suggesting no significant differences between prophylactic oxytocin given before versus after placental separation on PPH, need for additional uterotonic, or nausea/vomiting. The overall certainty of the evidence was mostly low or very low due to imprecision. Protocol: CRD42020186797.

## Introduction

Post-partum hemorrhage (PPH) is the leading cause of maternal mortality and an important cause of severe maternal morbidity worldwide [1–3]. The estimated incidence of PPH in women delivered by cesarean section (CS) is 3–15%, compared to 2–4% in those delivered vaginally [4, 5]. Rates of CS are increasing worldwide [6] and could be one of the factors associated with the increasing rates of PPH [3, 5].

Uterine atony is responsible for 50–80% of all cases of PPH [2, 3]. According to the World Health Organization (WHO), in settings where multiple uterotonic options are available, intravenous (IV) or intramuscular oxytocin is recommended for the prevention of PPH for all births [2]. However, there are no clear recommendations on the best time to administer oxytocin to prevent PPH in women delivered by CS. Available guidelines have various recommendations on doses, routes, and regimens for the administration of prophylactic oxytocin at CS, but most fail to provide any specific guidance on timing of administration [2, 7–11]. While some obstetricians give prophylactic oxytocin at various moments before fetal delivery at CS, others administer it soon after the infant is born and the umbilical cord is clamped, and yet others delay oxytocin administration until the placenta had detached from the uterus. The timing of oxytocin administration at CS can potentially affect the volume of maternal blood loss as well as the incidence of drug-related adverse effects which is especially relevant in this context since all women giving birth by cesarean are under regional or general anesthesia and receive several other drugs with cardiovascular effects [12, 13].

We identified several trials addressing this topic, but no previous systematic review. It is important to compile the best available evidence on the timing of oxytocin administration during CS to prevent PPH to optimize the care given to the growing number of women who deliver by this route.

The objectives of this systematic review were to identify, critically appraise, and synthesize the evidence on the effects of administrating prophylactic oxytocin at different times in women delivered by CS. We wanted to answer the following question: In women giving birth by CS, what are the effects of administrating prophylactic oxytocin at different moments on the incidence of PPH and associated outcomes, according to randomized controlled trials?

## Methods

The review followed the recommendations of the Cochrane Handbook for Systematic Reviews of Interventions [14] and was reported according to the PRISMA statement [15]. The review protocol was registered prospectively (CRD42020186797).

### Types of studies

Only randomized clinical trials (RCT) with a parallel design were eligible for inclusion. We included abstracts if they provided sufficient information to allow quality assessment.

### Types of participants

We included studies that recruited women of any age and race, with or without comorbidities, at low/average or high risk for PPH, with singleton or multiple pregnancies, who were submitted to a primary or repeat, pre-labor or intrapartum CS, for any indication and at any gestational age, with or without previous use of oxytocin for labor induction or augmentation in the index pregnancy. Studies that included participants having vaginal and cesarean deliveries were included only if data for CS was presented separately.

### Types of interventions

We included studies that compared the use of prophylactic oxytocin alone at different moments of administration during a CS: before fetal delivery versus after fetal delivery, or after fetal delivery but before placental separation/delivery versus after placental separation/delivery. Trials that used prophylactic oxytocin in any dose, route, or regimen, were eligible for inclusion in the review as long as these were similar in the comparator group. We excluded studies that compared oxytocin versus other pharmacological agents (alone or combined), placebo, or no intervention. We also excluded studies that used oxytocin associated with any other pharmacological agent to prevent PPH at CS.

### Outcomes

We included studies that reported at least one of our outcomes of interest. The selection of these outcomes was based on the list of PPH prevention core outcome set developed by the CROWN initiative [16]. Our three primary outcomes were PPH ≥ 1000 mL (measured by any method), need for additional uterotonics, and any immediate adverse effects of oxytocin, including headache, nausea/vomiting, flushing, hypotension (defined by study authors) and changes in cardiac rhythm (defined by study authors). Our secondary outcomes were volume of blood loss at CS, blood transfusion, shock, severe maternal morbidity (organ failure or coma or admission to ICU or hysterectomy), maternal transfer to a higher level of care, PPH-related maternal mortality, maternal satisfaction, and proportion of women breastfeeding at discharge. We assessed the primary outcomes at any time point within the first 24 hours of delivery.

### Search strategy

We developed a search strategy that was adapted and run in the following databases, without date, language, or publication status restrictions (S1 Table): CINAHL (Cumulative Index to Nursing and Allied Health Literature), Cochrane Library (via Wiley), Embase (via Elsevier), Global Index Medicus (via Biblioteca Virtual em Saúde—BVS), LILACS (Latin American and Caribbean Health Sciences Literature, via BVS), MEDLINE (via PubMed), SciELO, and two trial registry platforms (Clinicaltrials.gov and WHO International Clinical Trials Registry Platform-ICTRP). We also conducted a search for grey literature in Opengrey (https://opengrey.eu) and screened the reference lists of all included studies and relevant systematic reviews. We included all relevant studies identified from database inception until May 31, 2020.

### Process of study selection and data extraction

We uploaded all references retrieved into the Rayyan platform [17] and excluded duplicates. Two review authors independently assessed titles and abstracts, selected potentially eligible references for full text reading, extracted data, and assessed the quality (risk of bias) of each included trial. We used the Cochrane Risk of Bias (RoB) tool [14] to grade seven domains of each study (random sequence generation, allocation concealment, blinding of participants and personnel, blinding of outcome assessment, incomplete outcome data, selective reporting, and other source of bias) as being at high, low, or unclear risk of bias. We assessed the third, fourth and fifth domains at outcome-level. Any disagreements in the process of study selection, data extraction, and quality assessment were solved by a third reviewer. We contacted trial authors for missing data and additional information.

### Data analyses

We pooled results from similar studies using Review Manager 5.4 (The Cochrane Collaboration, 2020). We calculated risk ratios (RR) and mean differences (MD), and their respective 95% confidence intervals (CI), for dichotomous and continuous data, respectively. When a metanalysis was not possible, we present results descriptively. We conducted two main comparisons: i) prophylactic oxytocin given before fetal delivery versus after fetal delivery, and ii) oxytocin given after fetal delivery but before placental separation/delivery versus after placental separation/delivery. We carried out analyses for all outcomes on an intention-to-treat basis. We pooled data using random-effects metanalyses. We used Chi^2^ and I^2^ tests to assess statistical heterogeneity; I^2^ ≥ 50% was considered an indication of high heterogeneity. To investigate heterogeneity, we planned to conduct subgroup analyses for the primary outcomes (when data were available) according to baseline risk for PPH (low/regular x high risk). We did not conduct this analysis because statistical heterogeneity was low. We planned to conduct the following sensitivity analyses for the main comparisons and primary outcomes, when data were available: restricted only to high-quality studies (i.e. those with a ‘low risk of bias’ for random sequence generation and allocation concealment), and restricted only to studies that assessed blood loss objectively (e.g. by weighing surgical drapes/swabs or by using blood collecting devices). These analyses were not done because all studies for the first comparison were high-quality and measured blood loss objectively, and there was only one study for the second comparison. We planned to investigate publication bias by visual inspection of funnel plots for metanalysis with at least 10 studies but this was not possible due to the small number of trials.

We used the GRADE approach [18] to assess the quality (certainty) of the body of evidence (high, moderate, low or very low) for the two main comparisons (oxytocin given before versus after fetal delivery, and before versus after placental separation/delivery). The certainty of the evidence was downgraded due to trial limitations, inconsistency, indirectness, imprecision, and publication bias.

## Results

**Fig 1:**
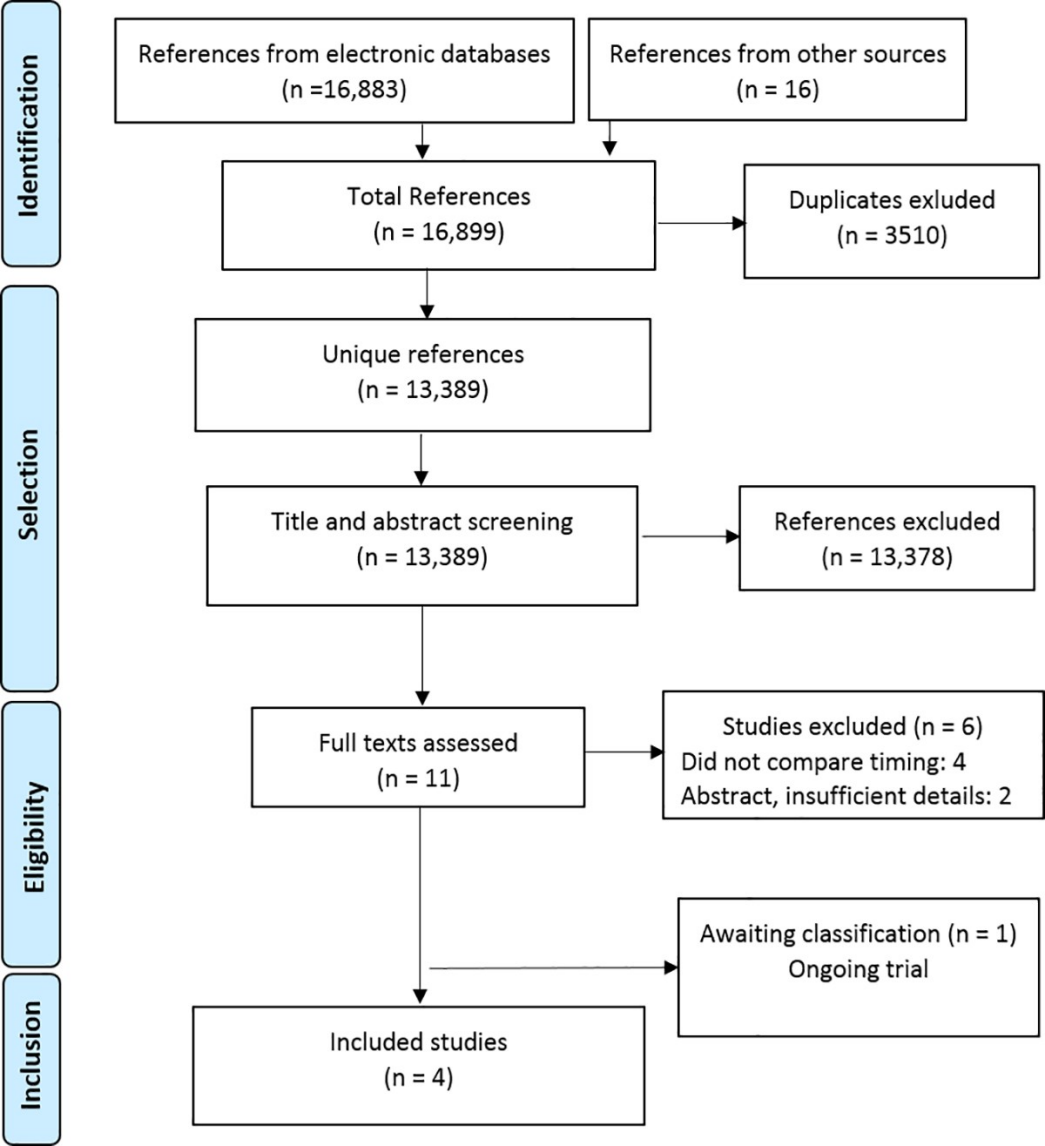
Flowchart of the process of study selection.

The electronic searches retrieved 16,883 references and the manual search added 16 references (Fig 1). After excluding 3510 duplicates, we screened 13,389 references and selected 11 publications for full text reading. We excluded four studies and two abstracts (S2 Table), identified one ongoing trial (S3 Table), and included four studies [19–22].

These four studies were conducted between 2012 and 2020, in Egypt [19, 22], India [20], and Turkey [21], and included a total of 701 women (Table 1 and S4 Table). None of the trials were double-blinded. Where this information was available, most or all study participants were at term, at low risk for PPH, undergoing a prelabour, primary CS, under spinal anesthesia (Table 1). Three trials [19, 21, 22] compared oxytocin given before versus after fetal delivery. Abdelaleem et al started a 4-hour intravenous (IV) oxytocin infusion with 30 IU/500 ml 0.9% saline immediately after incision of the visceral peritoneum compared to immediately after fetal delivery and umbilical cord clamping [19]. Takmaz et al performed the same comparison but used a different dose (20 IU/500 ml 0.9% saline) [21]. Tharwat et al started a 15-minute IV drip with 10 IU/200 ml Ringer lactate during induction of anesthesia, before skin incision, compared to after delivery of the fetus [22]. Mangla et al injected oxytocin (5 IU/10 ml saline) directly into the myometrium after the fetus was delivered, either before (n = 50) or after (n = 50) placental separation [20]. The four studies assessed the need for additional uterotonics; two studies [20, 22] assessed PPH and adverse effects (nausea and vomiting). All four studies reported objectively measured intra-operative blood loss; two studies [19, 21] reported blood transfusions and hysterectomy. None of the trials reported any of our other secondary outcomes (S4 and S5 Tables).

**Table 1:** Main characteristics of trials on timing of oxytocin administration at cesarean.

	Abdelaleem 2018 [19]	Takmaz 2020 [21]	Tharwat 2020 [22]	Mangla 2012 [20]
Setting	Egypt, 3^ary^ university hospital	Turkey, university hospital	Egypt, university hospital	India, 3^ary^ university hospital
Period of data collection	2016–2017	2019	2016	Unclear (pre-2012)
Sample size	200	101	300	100
Gestational age	all term	all term	all term	no information
Baseline risk for PPH	all low risk	all low risk	all low risk	no information
Parity	40% nulliparas	85% nulliparas	unclear	no information
	60% multiparas	15% multiparas		
Participants with previous CS	27.5%	0%	32%	no information
Type of CS	Pre-labor, scheduled	Pre-labor, scheduled	Pre-labor, scheduled	no information
Previous exposure to oxytocin (induction/ augmentation)	not applicable	not applicable	not applicable	no information
Anesthesia	spinal	spinal	spinal	spinal or general
Oxytocin route and regimen	IV	IV	IV	Intra-myometrial
Total dose, total duration	30 IU, 4h	20 IU, 4h	10 IU, 15 min	5 IU, seconds
Dose/diluent, speed of administration, Infusion rate (IU/min)	30 IU/500 ml 0.9% saline, 125 ml/h, Rate: 0.125 IU/min	20 IU/500 ml 0.9% saline, 125 ml/h, Rate: 0.083 IU/min	10 IU/200 ml Ringer, Rate:0.665 IU/min	5 IU/10 ml 0.9% saline, 5 ml injected in each cornu
Timing of administration	Infusion started immediately after incision of visceral peritoneum x immediately after umbilical cord clamping	Infusion started immediately after incision of visceral peritoneum x immediately after umbilical cord clamping	Drip started before skin incision x after fetal delivery	Myometrial injection given after fetal delivery before x after placental separation
Comparison	Before x After Fetal delivery	Before x After Fetal delivery	Before x After Fetal delivery	Before x After Placental delivery
Outcomes reported	Additional uterotonic	Additional uterotonic	PPH > 1000 mL	PPH ≥ 1000 mL
	Total blood loss volume	Total blood loss volume	Additional uterotonic	Additional uterotonic
	Blood transfusion	Blood transfusion	Adverse effects	Adverse effects
	Hysterectomy	Hysterectomy	Total blood loss volume	Total blood loss volume

CS: cesarean section, IU: international units, IV: intravenous, min: minute, PPH: post-partum hemorrhage.

**Fig 2:**
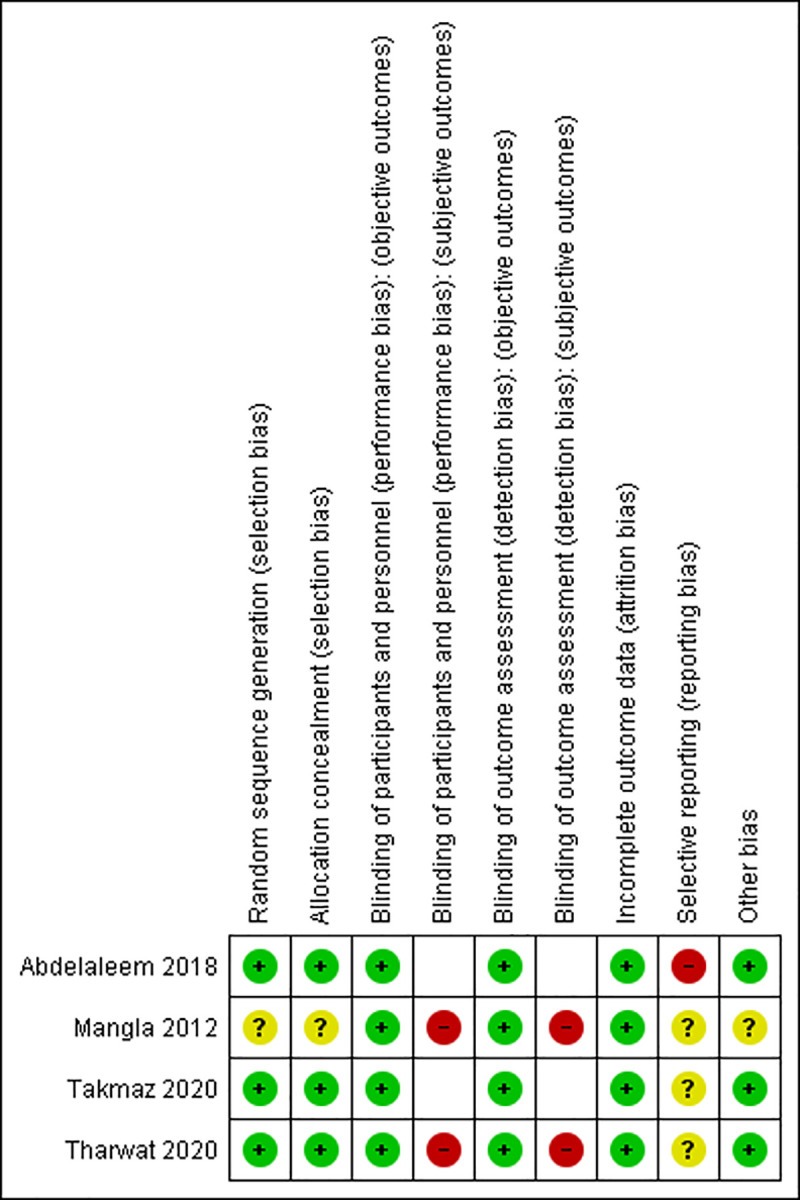
Risk of bias summary.

Fig 2 summarizes the risk of bias of the included trials (see S6 Table for details). Three studies [19, 21, 22] had low risk of bias for random sequence generation and allocation concealment; the third study had unclear risk of bias for these domains due to missing information (authors did not reply to our contacts). None of the trials were double-blinded. All studies had at least one domain with unclear or high risk of bias.

### Comparison 1. Prophylactic oxytocin given before fetal delivery versus after fetal delivery

Three RCTs including a total of 601 participants assessed the effects of administrating oxytocin before versus after fetal delivery at CS. All participants had a low/regular baseline risk for PPH and received intravenous (IV) oxytocin infusions [19, 21, 22]. We conducted subgroup analyses because there was an important difference in the timing of administration of oxytocin before fetal delivery: one trial (22) started oxytocin at skin incision while the other two trials [19, 21] started the infusion immediately before uterine incision.

#### Incidence of PPH

Only Tharwat et al 2020 assessed this outcome in a study involving 300 women submitted to elective, pre-labor CS at term [22]. There was no significant difference in the incidence of PPH between women who received a short (15-minute) oxytocin drip (10 IU/200 ml Ringer lactate, 0.665 IU/min) initiated at anesthesia induction before skin incision versus after fetal delivery (3/150 versus 5/150; RR 0.60, 95% CI 0.15 to 2.47; 1 RCT, 300 participants, low certainty evidence) (S7 Table).

#### Need for additional uterotonic

**Fig 3:**
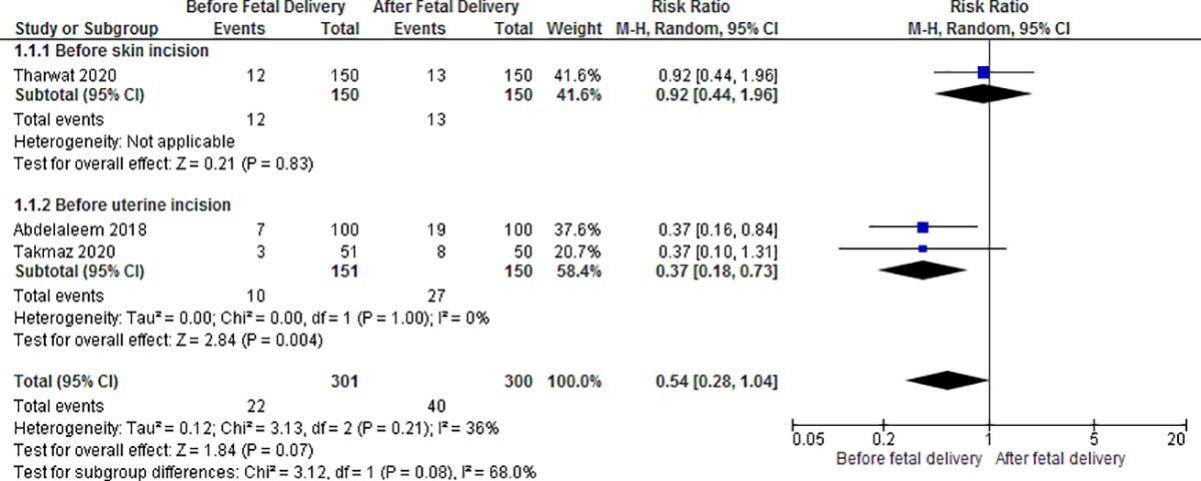
Forest plot of oxytocin given before versus after fetal delivery. Outcome: need for additional uterotonic.

The pooled estimate of the three trials [19, 21, 22] showed no statistically significant difference in the need for additional uterotonics when oxytocin was given before versus after fetal delivery (22/301 versus 40/300; RR 0.54, 95% CI 0.28 to 1.04; I^2^ = 36%; 3 RCTs, 601 participants; moderate certainty evidence) (S7 Table and Fig 3). In the subgroup analysis, there was no significant difference in the need for additional uterotonics when the oxytocin infusion was started before skin incision versus after fetal delivery (12/150 versus 13/150; RR 0.92, 95% CI 0.44 to 1.96; 1 RCT, 300 participants). However, when the infusion was started immediately before uterine incision versus after fetal delivery, earlier administration of oxytocin was associated with a significant reduction in the need for additional uterotonics (10/151 versus 27/150; RR 0.37, 95% CI 0.18 to 0.73; I^2^ = 0%; 2 RCTs, 301 participants).

#### Adverse effects of oxytocin

Only one trial reported the incidence of nausea and/or vomiting [22]. There was no significant difference between women who received oxytocin before or after fetal delivery (23/150 versus 19/150; RR 1.21, 95% CI 0.69 to 2.13; 1 RCT, 300 participants, low certainty evidence) (S7 Table).

Two studies provided information on participants´ blood pressure and heart rate (HR) measures but authors did not define hypotension or tachycardia. Abdelaleem et al reported no significant changes in participants´ mean HR, systolic blood pressure (SBP), or diastolic blood pressure (DBP) measured immediately before and after the surgery within groups or between groups [19]. Tharwat et al reported that SBP and DBP decreased, and HR increased immediately after surgery in both groups, but that these changes were less intense in the women who started oxytocin infusion before skin incision than after fetal delivery [22].

#### Volume of blood loss

**Fig 4:**
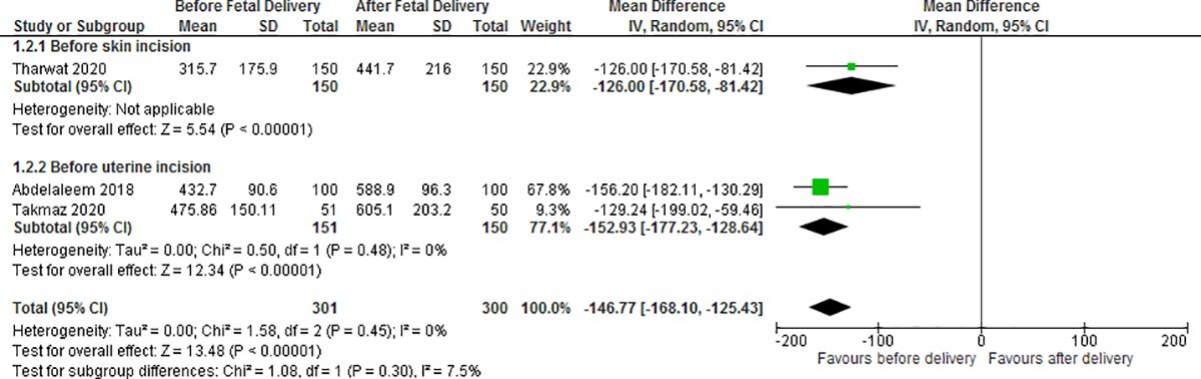
Forest plot of oxytocin given before versus after fetal delivery. Outcome: volume of blood loss.

The pooled estimate of three trials [19, 21, 22] showed a significant reduction in blood loss during CS of approximately 150 mL in the group that started IV oxytocin infusion before fetal delivery (MD -146.77 mL, 95% CI -168.10 to -125.43, I^2^ = 0%, 3 RCTs, 601 participants, moderate certainty evidence) (Fig 4 and S7 Table).

#### Blood transfusion

**Fig 5:**
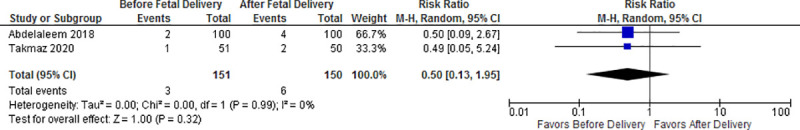
Forest plot of oxytocin given before versus after fetal delivery. Outcome: incidence of blood transfusion.

The pooled estimate from two trials showed no statistically significant differences between groups for this outcome (3/151 pre-fetal delivery versus 6/150 post-fetal delivery; RR 0.50, 95% CI 0.13 to 1.95; I^2^ = 0%; 2 RCTs, 301 participants, low certainty evidence) (Fig 5 and S7 Table).

#### Severe maternal morbidity

**Fig 6:**
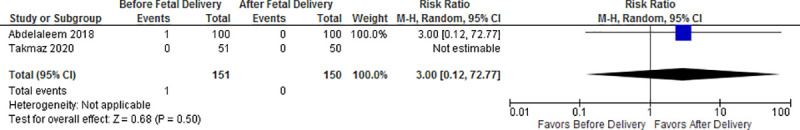
Forest plot of comparison: Pre-fetal extraction versus post-fetal extraction. Outcome: incidence of hysterectomy.

Two trials [19, 21] reported the incidence of hysterectomy among participants. The was no significant difference between the groups that received oxytocin before or after fetal delivery but the effect estimate was highly imprecise due to the very low number of events (1/151 versus 0/150; RR 3.00; 95% CI 0.12 to 72.77; I^2^ = 0%; 2 RCTs, 301 participants, low certainty evidence) (Fig 6 and S7 Table).

### Comparison 2. Prophylactic oxytocin given after fetal delivery, before placental separation/delivery versus after placental separation/delivery

Only one trial (100 participants) assessed this comparison in women who received intra-myometrial oxytocin before placental separation (n = 50) or after placental separation (n = 50) [20]. There was no information on participants´ baseline risk for PPH, parity, gestational age, or type of CS. The authors did not reply to our requests for additional information.

#### Incidence of PPH

Mangla et al 2012 reported that there were no cases of blood loss ≥ 1000 ml in the two groups (0/50 versus 0/50, very low certainty evidence), (S8 Table).

#### Need for additional uterotonic

The authors also reported that no women in both group required additional uterotonics (0/50 versus 0/50, very low certainty evidence) (S8 Table).

#### Adverse effects of oxytocin

There was no statistically significant difference in the incidence of nausea or vomiting requiring additional antiemetics between the groups (1/50 versus 1/50, RR 1.00; 95% CI 0.06 to 15.55; 100 participants; 1 RCT, very low certainty evidence) (S8 Table). The authors did not define hypotension but measured changes in SBP every 5 minutes (for 15 minutes) after oxytocin injection; compared to baseline measurements, the rate of women with a decrease in SBP of 6 to 10 mm Hg was similar in both groups (36/50 versus 39/50).

#### Volume of blood loss

The women who received oxytocin before placental separation had lower mean blood loss than those who received it after placental separation (412 mL versus 460 mL, respectively). However, we could not calculate mean differences and 95% CIs because the authors did not provide standard deviations, and did not reply to our requests.

## Discussion

Despite our comprehensive search, we identified only four trials that assessed the effects of giving prophylactic oxytocin at different moments during a CS. None of the studies were randomized double-blinded placebo-controlled trials. Three trials provided data for the comparison of IV oxytocin administration before versus after fetal delivery; there were no significant differences between groups for PPH, nausea/vomiting, blood transfusion or hysterectomy. However, mean intra-operative blood loss was significantly lower in women who received oxytocin before compared to after fetal delivery, and the need for additional uterotonics was lower when oxytocin infusion was started immediately before uterine incision rather than after fetal delivery. Only one trial provided data on prophylactic oxytocin (administered into the myometrium) before versus after placental separation, and found no significant differences between groups for PPH, need for additional uterotonic or nausea/vomiting. The overall certainty of the evidence was mostly low or very low due mainly to imprecision.

Overall, the limited existing evidence suggests that earlier administration of prophylactic oxytocin at CS may be somewhat more beneficial than later administration (i.e., after fetal delivery), without an increase in adverse effects. Most studies included only healthy women at low risk for PPH undergoing elective, pre-labor CS at term, under spinal anesthesia. Therefore, these findings cannot be generalized to all women giving birth by CS. While participants´ characteristics were mostly homogeneous, the oxytocin regimens varied between studies. The three studies that compared administration before versus after fetal delivery [19, 21, 22] used IV infusions with similar oxytocin concentrations (0.04 to 0.06 IU/ml) but the infusion rate ranged from 0.083 IU/min [21] to 0.665 IU/min [22], an eightfold difference. The total oxytocin dose and duration of administration also varied between studies, from 10 IU in 15 minutes [21] to 30 IU in 4 hours [19]. This can have important clinical implications since the dose of oxytocin infused influences oxytocin plasma levels in a dose dependent way [23, 24]. This could lead to differences in the effects of the drug on uterine contraction, and consequent volume of blood loss, as well as adverse effects. Finally, the moment of IV oxytocin administration before fetal delivery also varied. While two studies [19, 21] started a long-term (4-hour) infusion immediately before delivery (after incision of visceral peritoneum), the third trial [22] started a short-term (15-minute) infusion at least 5–10 minutes before fetal delivery (before skin incision, at induction of anesthesia). This can influence the effectiveness of the medication, since pharmacokinetic studies indicate that IV synthetic oxytocin starts to act within very few minutes of injection and has a half-life of about 15–30 minutes [25, 26].

Although IV oxytocin has been associated with important cardiovascular side effects that can lead to maternal death [12, 13, 27], only one [22] of the three trials that used endovenous oxytocin reported the incidence of nausea/vomiting (a possible reflex of hypotension), and mean changes in blood pressure and HR between the groups. This is a serious gap, since all women having a CS are under anesthesia and, therefore, have an increased risk for hypotension and changes in cardiac rhythm, especially if they have comorbidities [27–30].

Previous reviews have assessed the effects of various uterotonics, including oxytocin, to prevent PPH at CS compared to placebo or other uterotonics in different doses/regimens, and routes of administration [31–33]. However, to the best of our knowledge, this is the first systematic review to assess different timings of oxytocin administration to prevent PPH in women giving birth by CS. Strong points of the review include its comprehensive literature search, including grey literature, without language restrictions, its strict adherence to standard Cochrane methods including rigorous assessment of study quality and grading the certainty of the evidence [14]. Limitations of the review include the small number of included trials, the limited success in obtaining additional information from study authors on patient characteristics and methodological details, clinical heterogeneity in the regimens used to administer oxytocin, and the lack of important primary outcome measures, including adverse effects, in several studies. Moreover, the overall certainty of the evidence was mostly low or very low due to imprecision.

There is a need for additional, well conducted and well reported, trials on the timing of prophylactic oxytocin in women giving birth by CS, to increase the overall certainty of the evidence on this important clinical question. Ideally, future RCTs should be placebo controlled and double-blinded, involve other obstetric populations (women with previous CS and those at high risk for PPH), as well as other types of CS (in the 1^st^ and 2^nd^ stages of spontaneous and induced labor previously exposed to oxytocin), and measure all PPH prevention core outcomes, including adverse effects and women´s views [16].

## Conclusions

This systematic review identified only four randomized trials that assessed the effectiveness and safety of prophylactic oxytocin given at different moments during a CS. In women submitted to pre-labor CS under regional anesthesia, there is limited evidence from three trials indicating no significant differences between IV administration of prophylactic oxytocin before versus after fetal delivery on PPH, nausea/vomiting, blood transfusion, or hysterectomy. However, limited evidence suggests that IV administration of oxytocin shortly before fetal delivery may reduce blood loss and the need for additional uterotonics. There is very limited evidence, from a single trial, suggesting no significant differences between prophylactic oxytocin given before versus after placental separation on PPH, need for additional uterotonic, or nausea/vomiting. The overall certainty of the evidence was mostly low or very low due to imprecision. Therefore, more high quality, and well reported, trials are needed on this important clinical question.

## Supporting information

S1 Checklist PRISMA checklist. (PDF)

S1 Table Search strategy. (PDF)

S2 Table Excluded studies. Reasons for exclusion of studies selected for full text reading.
(PDF)

S3 Table Ongoing trials. Characteristics of ongoing trials.
(PDF)

S4 Table Study details. Details of included studies.
(PDF)

S5 Table Postpartum hemorrhage core outcome sets. Core outcomes reported in included trials.
(PDF)

S6 Table Risk of bias. Judgments and justifications for risk of bias assessments.
(PDF)

S7 Table Summary of findings 1. Summary of findings table and GRADE for comparison 1: Prophylactic oxytocin administered before versus after fetal delivery at cesarean section.
(PDF)

S8 Table Summary of findings 2. Summary of findings table and GRADE for comparison 2: Prophylactic oxytocin administered before versus after placental separation.
(PDF)

S1 Text Study protocol. (PDF)
