# Supplementary material for: Timing of oxytocin administration to prevent post-partum hemorrhage in women delivered by cesarean section: A systematic review and metanalysis
Source: PLoS One. 2021 Jun 3;16(6):e0252491. doi: 10.1371/journal.pone.0252491 (PMC8174699; doi:10.1371/journal.pone.0252491)
Supplement: S2 Table — Reasons for exclusion of studies selected for full text reading. (PDF) [file pone.0252491.s003.pdf]

S2 Table. Excluded studies with reasons

	Study	Reason for exclusion
1	**Garza-Hinojosa 2015**<br>Garza-Hinojosa A, González-Cordero G. Cambios hemodinámicos y electrocardiográfi cos con el uso de oxitocina en bolo durante la cesárea. Ver Mex Anest. 2015;38(S1): S257-62.	Did not compare different timings
2	**Guerra 2005**<br>Guerra GB. Cesárea electiva. Efecto de la oxitocina endovenosa en la perdida hemática transoperatoria. Rev Obstet Ginecol Venez. 2005;65(2).	Did not compare different timings
3	**Phaswana 2016**<br>Phaswana VY. A Randomized Double Blinded Study Comparing The Haemodynamic Effects of Intravenous Bolus of 3 IU vs. 5 IU Oxytocin During Caesarean Section Delivery Under Spinal Anaesthesia at Dr. George Mukhari Academic Hospital. Research report submitted to the faculty of Medicine at University of Limpopo (Medunsa Campus) in partial fulfilment for the degree of Master of Medicine in Anaesthesiology. 2016	Did not compare different timings
4	**Singh 2015** (Abstract)<br>Singh KP, Kameshore N, Kamei H. Prophylactic intramuscular injection of oxytocin vs intravenous infusion of oxytocin to minimize blood loss at caesarean section. Int J Gynecol Obstet. 2015;131(Suppl. 5): E72–E313.	Insufficient information to assess outcome measures and study quality
5	**Schaefer 2004** (Abstract)<br>Schaefer A, Klein L, Wolfe P, Heindricks G, Downs L, Guinn D. Double blind RCT of early versus traditional oxytocin management in the third stage to prevent blood loss [abstract]. American Journal of Obstetrics and Gynecology 2004;191 (6 Suppl 1):S69.	Did not provide data separately for CS
6	**Zarzur 1992**<br>Zarzur E. A Ocitocina e a Operação Cesariana. Rev Bras Anest. 1992;42(4):293-5.	Did not compare different timings
