# Supplementary material for: Timing of oxytocin administration to prevent post-partum hemorrhage in women delivered by cesarean section: A systematic review and metanalysis
Source: PLoS One. 2021 Jun 3;16(6):e0252491. doi: 10.1371/journal.pone.0252491 (PMC8174699; doi:10.1371/journal.pone.0252491)
Supplement: S3 Table — Characteristics of ongoing trials. (PDF) [file pone.0252491.s004.pdf]

S3 Table. Characteristics of ongoing trials

Study	Status	Date of registration	Design	Participants	Intervention	Comparator	Main outcomes	Funding
PACTR201909775190644<br><br>"A randomized controlled trial of two methods of oxytocin infusion administration in prevention of blood loss at caesarean section".	Completed, no results available	05 September 2019	RCT, double-blind	• 20-40 years<br>• singleton pregnancy<br>• >37 weeks<br>• ASA 1 or 2<br>• n = 230	30 IU in 500 mL of 0.9% saline commenced just before incision on the uterus and infused at 125 ml/h	30 IU in 500 mL of 0.9% saline after umbilical cord clamping and infused at 125 ml/h	• Blood loss during CS<br>• Post-operative blood loss<br>• Difference in pre- and post-operative packed cell volume/ hemoglobin concentration<br>• Need for additional uterotonics<br>• Need for blood transfusion<br>• Need for additional surgical procedures	none

CS: Cesarean section, RCT: randomized controlled trial
