# Supplementary material for: Timing of oxytocin administration to prevent post-partum hemorrhage in women delivered by cesarean section: A systematic review and metanalysis
Source: PLoS One. 2021 Jun 3;16(6):e0252491. doi: 10.1371/journal.pone.0252491 (PMC8174699; doi:10.1371/journal.pone.0252491)
Supplement: S4 Table — Details of included studies. (PDF) [file pone.0252491.s005.pdf]

**S4 Table.** Details of included studies

| Abdelaleem 2018 | |
|---|---|
| **Method** | single blind RCT<br>protocol: NCT03344302 |
| **Participants** | Setting: women managed at Assiut University (tertiary teaching hospital), Egypt<br>Dates of recruitment: October 2016- August 2017<br>**Inclusion criteria:** 200 women, 20-40 years, primigravida or multigravida, singleton pregnancy, 37-42 weeks, ASA status I or II, booked for elective CS.<br>**Exclusion criteria:** women at high risk for uterine atony (multiple gestation, placenta previa, polyhydramnios, fetal macrosomia) and two or more previous CS, pre-eclampsia, current or previous history of heart disease, liver, renal disorders or known coagulopathy, anemia. |
| **Interventions** | **Group 1**: IV infusion of oxytocin (Syntocinon®; Novartis Pharma, Berne, Switzerland) 30 IU/ 500 mL 0.9% saline at a rate of 125 mL/h started immediately after opening the visceral peritoneum and just before incising the lower uterine segment. (n=100 randomized)<br>**Group 2**: IV infusion of oxytocin (Syntocinon®; Novartis Pharma, Berne, Switzerland) 30 IU/ 500 mL 0.9% saline at a rate of 125 mL/h started immediately after clamping the umbilical cord.<br>(n=100 randomized) |
| **Outcomes** | Volume of blood loss during CS (measured by adding the volume of the suction bottle with the blood soaked sponges-known dry weight).<br>Additional uterotonics. Blood transfusion.<br>Additional surgical procedures (uterine artery ligation, hemostatic sutures or peripartum hysterectomy) |
| **Notes** | Funding: not reported<br>Conflict of interest: none |

| Mangla 2012 | |
|---|---|
| **Method** | RCT<br>No protocol. |
| **Participants** | Setting: Guru Gobind Singh Hospital, Jamnagar, Gujarat, India<br>Dates of recruitment: No information<br>**Inclusion criteria:** "women operated by lower segment cesarean section". No other information<br>**Exclusion criteria:** No information |
| **Interventions** | **Group 1**: IV infusion 20 IU/500 ml Ringer after placental detachment, no information on rate or duration of infusion<br>(n=50 randomized). This group was not included in the SR because of different route of administration.<br>**Group 2**: Intra-myometrial 5 IU in 10 cc normal saline; 5 cc injected in each cornu of the uterus after separation of placenta.<br>(n=50 randomized)<br>**Group 3**: Intra-myometrial 5 IU in 10 cc normal saline; 5 cc injected in each cornu of the uterus before separation of placenta.<br>(n=50 randomized) |
| **Outcomes** | Volume of blood loss during CS and 1$^{st}$ hour after (measured by adding volume in graduated suction jar and number of soaked sponges-no information on how loss was measured in the first hour after CS). Additional uterotonic. Nausea/vomiting requiring antiemetic. |
| **Notes** | Funding: none<br>Conflict of interest: none |

| Takmaz 2020 | |
|---|---|
| **Method** | RCT<br>protocol: NCT03967171 |
| **Participants** | Setting: women managed at  Department of Obstetrics and Gynecology of Bezmialem University, Turkey<br>Dates of recruitment: April-June 2019<br>**Inclusion criteria:** 101 women,  18-40 years, primary elective CS under spinal anesthesia at term (> 37 weeks) with ASA status I or II.<br>**Exclusion criteria:** uterine over distention (suspected macrosomia, multiple pregnancies, polyhydramnios); previous history of some medical conditions, including moderate to severe hypertension, diabetes mellitus, or any blood and thrombophilia disorders; emergency surgery because of placenta previa or placental abruption; anticoagulation therapy; or a history of other major abdominal surgeries. |
| **Interventions** | **Group 1**: IV oxytocin infusion (Synpitan forte®; Deva Pharma, Istanbul, Turkey) consisting of 20 IU in 500 mL of normal 0.9 % saline at 125 mL/h started  immediately after incision of the visceral peritoneum.<br>(n=51 randomized)<br>**Group 2**: IV oxytocin infusion (Synpitan forte®; Deva Pharma, Istanbul, Turkey) consisting of 20 IU in 500 mL of normal 0.9 % saline at 125 mL/h started  immediately after umbilical cord clamping.<br>(n=50 randomized) |
| **Outcomes** | Volume of blood loss during CS (measured by adding the volume of the suction bottle with the blood soaked sponges-known dry weight).<br>Additional uterotonics. Blood transfusion.<br>Additional surgical procedures (uterine artery ligation, hemostatic sutures or peripartum hysterectomy) |
| **Notes** | Funding: not reported<br>Conflict of interest: none<br>All CS were done by the same two physicians. |

| Tharwat 2020 | |
|---|---|
| **Method** | RCT<br>no protocol |
| **Participants** | Setting: women managed at Ain-Shams University Maternity Hospital, Cairo-Egypt<br>Dates of recruitment: March -September 2016<br>**Inclusion criteria:** 300 women, 20-35 years, BMI 20 kg/m$^2$ - 35 kg/m$^2$, Hb ≥ 10 g/dl, gestational age ≥ 37 weeks, elective CS.<br>**Exclusion criteria:** bleeding disorders, intake of steroids, anticoagulant and anti-platelets drugs, multiple pregnancy, pregnancy induced hypertension, macrosomia, polyhydramnios, antepartum hemorrhage, placenta previa, any complication during the surgery, history of postpartum hemorrhage, medical disorders (e.g., hypertension, diabetes, heart diseases) and contraindication to oxytocin infusion (e.g., hypersensitivity). |
| **Interventions** | **Group 1**: IV oxytocin drip 10 IU in 200 ml Ringer over 15 minutes (250 drops/minute) starting before skin incision<br>(n=150 randomized)<br>**Group 2**: IV oxytocin drip 10 IU in 200 ml Ringer over 15 minutes (250 drops/minute) starting after fetal delivery<br>(n=150 randomized) |
| **Outcomes** | Postpartum hemorrhage. Additional uterotonics. Any adverse effects. Volume of blood loss during CS (measured by adding the volume of the suction bottle with the blood soaked sponges- known dry weight). |
| **Notes** | Funding: not reported<br>Conflict of interest: not reported<br>Placenta delivered by controlled cord traction, two layer closure of the uterine incision, and surgeons avoided delivering the uterus for suturing unless clinically indicated. |

BMI: body mass index, CS: cesarean section, IU: international units, IV: intravenous, RCT: randomized controlled trial
