# Supplementary material for: Timing of oxytocin administration to prevent post-partum hemorrhage in women delivered by cesarean section: A systematic review and metanalysis
Source: PLoS One. 2021 Jun 3;16(6):e0252491. doi: 10.1371/journal.pone.0252491 (PMC8174699; doi:10.1371/journal.pone.0252491)
Supplement: S5 Table — Core outcomes reported in included trials. (PDF) [file pone.0252491.s006.pdf]

S5 Table. Postpartum hemorrhage core outcome sets* reported in included trials

	Outcome	Measure (in each group)	Study			
			Abdelaleem 2018	Takmaz 2020	2020	Mangla 2012
1	Blood loss (from birth up to cessation of active bleeding)					
	≥ 1000 ml	N women with this loss	no	no	yes	yes
	total volume	mean or median volume	yes	yes	yes	yes
2	Shock (defined by trialists)	N women	no	no	no	no
3	Maternal death					
	PPH-related maternal deaths	N women	no	no	no	no
	all cause mortality	N women	no	no	no	no
4	Blood transfusion					
	Wm receiving any RBC product	N women	yes	yes	No	No
	total volume	mean or median RBC units	no	no	No	No
5	Transfer to higher level of care	N women	No	No	No	No
6	Use of additional uterotonics	N women	yes	yes	yes	yes
7	Adverse effects (defined by trialists)	N women	no	no	Yes (nausea /vomiting)	Yes (nausea/ vomiting)
8	Breastfeeding	N women	no	no	no	no
9	Patient reported outcomes	N women	no	no	no	no

N: number, PPH: post-partum hemorrhage, RBC: red blood cell

*Source: Meher S, Cuthbert A, Kirkham JJ, et al. Core outcome sets for prevention and treatment of postpartum haemorrhage: an international Delphi consensus study. BJOG. 2019 Jan;126(1):83-93.
