# Supplementary material for: Timing of oxytocin administration to prevent post-partum hemorrhage in women delivered by cesarean section: A systematic review and metanalysis
Source: PLoS One. 2021 Jun 3;16(6):e0252491. doi: 10.1371/journal.pone.0252491 (PMC8174699; doi:10.1371/journal.pone.0252491)
Supplement: S6 Table — Judgments and justifications for risk of bias assessments. (PDF) [file pone.0252491.s007.pdf]

S6 Table. Judgments and justifications for risk of bias assessments

Domain	Abdelaleem 2018		
1	Sequence generation	LOW	*"A statistician, not otherwise involved in the study, prepared a computer generated randomization table using Microsoft office Excel 2013 (Microsoft Corporation, Redmond, WA)"*
2	Allocation concealment	LOW	*"...and placed the allocation data in serially numbered closed opaque envelopes. Each envelope was labelled with a card noting the intervention type inside. The envelopes were opened only by the obstetrician according to the order of delivery of women. Allocation was never changed after opening the envelopes."*
3	Blinding participants/personnel (objective outcomes)	LOW	Blinding of participants was unconfirmed. Objective outcomes are unlikely to be influenced by this fact.
3	Blinding participants/personnel (subjective outcomes)	NA	There were no subjective outcomes.
4	Blinding outcome assessors (objective outcomes)	LOW	Blinding of outcome assessors was unconfirmed. Objective outcomes are unlikely to be influenced by this fact.
4	Blinding outcome assessors (subjective outcomes)	NA	There were no subjective outcomes.
5	Incomplete outcome	LOW	All randomized participants completed the study.
6	Selective reporting	HIGH	Study protocol (NCT03344302) was registered retrospectively. None of the secondary outcomes were pre-specified.
7	Other bias	LOW	There are probably no other sources of bias.
Domain	Mangla 2012		
1	Sequence generation	UNCLEAR	*"Women were divided randomly into three groups according to route of oxytocin administration during cesarean section".* No information on random sequence generation
2	Allocation concealment	UNCLEAR	No information on allocation concealment
3	Blinding participants/personnel (objective outcomes)	LOW	Blinding of participants was unconfirmed. However, objective outcomes are unlikely to be influenced by this fact.
3	Blinding participants/personnel (subjective outcomes)	HIGH	Blinding of participants and personnel was unconfirmed. Subjective outcomes are likely to be influenced by this fact.
4	Blinding outcome assessors (objective outcomes)	LOW	Blinding of outcome assessors was unconfirmed. However, the judgment of objective outcomes is unlikely to be influenced by this fact.
4	Blinding outcome assessors (subjective outcomes)	HIGH	Blinding of outcome assessors was unconfirmed. Subjective outcomes are likely to be influenced by this fact.
5	Incomplete outcome	LOW	Outcome data of all randomized participants were presented
6	Selective reporting	UNCLEAR	No study protocol.
7	Other bias	UNCLEAR	No information on baseline characteristics.

Domain	Takmaz 2020		
1	Sequence generation	LOW	*"The patients were divided into two groups by random allocation using a computer-generated random number table according to the timing of the IV infusion of oxytocin".*
2	Allocation concealment	LOW	Answer to email contact: *"The allocation numbers were put in concealed, opaque envelopes to conceal the outcomes of the randomisation. Opaque envelopes containing group assignments ensured blinding of study investigators, surgical teams and patients. The envelope was opened before surgery by an anesthesiologist in the operating room and oxytocin administrated according to randomisation by anesthesiologist."*
3	Blinding participants/personnel (objective outcomes)	LOW	Blinding of participants was unconfirmed and the interventions regimen were different. However, objective outcomes are unlikely to be influenced by this fact.
3	Blinding participants/personnel (subjective outcomes)	NA	There were no subjective outcomes.
4	Blinding outcome assessors (objective outcomes)	LOW	Answer to email contact: *"Outcome evaluations were carried out by researchers (SHI, BNAH) who were masked to the allocation status until the end of the project."*
4	Blinding outcome assessors (subjective outcomes)	NA	There were no subjective outcomes.
5	Incomplete outcome	LOW	All randomized participants completed the study.
6	Selective reporting	UNCLEAR	Study protocol (NCT03967171) was registered retrospectively. All planned outcomes analyzed.
7	Other bias	LOW	There are probably no other source of bias.
Domain	Tharwat 2020		
1	Sequence generation	LOW	*"Candidates were allocated in two groups, experimental arm (Group A) and control arm (Group B), according to a sequence of random numbers generated by computer with ratio 1:1."*
2	Allocation concealment	LOW	*"The assignment was done through opaque, sealed envelopes opened at the moment the mother enters the operative theatre."*
3	Blinding participants/personnel (objective outcomes)	LOW	Blinding of participants was unconfirmed and the interventions regimen were different. However, objective outcomes are unlikely to be influenced by this fact.
3	Blinding participants/personnel (subjective outcomes)	HIGH	Blinding of participants and personnel was unconfirmed and the interventions regimen were different. The subjective outcomes are likely to be influenced by this fact.
4	Blinding outcome assessors (objective outcomes)	LOW	Blinding of participants was unconfirmed and the interventions regimen were different. However, objective outcomes are unlikely to be influenced by this fact.
4	Blinding outcome assessors (subjective outcomes)	HIGH	Blinding of participants and personnel was unconfirmed and the interventions regimen were different. The subjective outcomes is likely to be influenced by this fact.
5	Incomplete outcome	LOW	Outcome data of all randomized participants were presented.
6	Selective reporting	UNCLEAR	No study protocol.
7	Other bias	LOW	There are probably no other source of bias.
