# Supplementary material for: Timing of oxytocin administration to prevent post-partum hemorrhage in women delivered by cesarean section: A systematic review and metanalysis
Source: PLoS One. 2021 Jun 3;16(6):e0252491. doi: 10.1371/journal.pone.0252491 (PMC8174699; doi:10.1371/journal.pone.0252491)
Supplement: S7 Table — Summary of findings table and GRADE for comparison 1: Prophylactic oxytocin administered before versus after fetal delivery at cesarean section. (PDF) [file pone.0252491.s008.pdf]

S7 Table. Summary of findings table and GRADE: prophylactic oxytocin administered before versus after fetal delivery at cesarean section

Summary of findings:

Prophylactic oxytocin administered Before versus After Fetal Delivery at cesarean section

Patient or population: women giving birth by cesarean section
Setting: hospital
Intervention: oxytocin given Before Fetal Delivery
Comparison: oxytocin given After Fetal Delivery

Outcomes	**Anticipated absolute effects*** (95% CI)		Relative effect (95% CI)	№ of participants (studies)	Certainty of the evidence (GRADE)	Comments
	**Risk with oxytocin given After Fetal Delivery**	**Risk with oxytocin given Before Fetal Delivery**				
Incidence of PPH ≥ 1000 mL	33 per 1.000	**20 per 1.000** (5 to 82)	**RR 0.60** (0.15 to 2.47)	300 (1 RCT)	⨁⨁◯◯ LOW [a]	
Need for additional uterotonic	133 per 1.000	**72 per 1.000** (37 to 139)	**RR 0.54** (0.28 to 1.04)	601 (3 RCTs)	⨁⨁⨁◯ MODERATE [b]	
Adverse effects of oxytocin – nausea/vomiting	127 per 1.000	**153 per 1.000** (87 to 270)	**RR 1.21** (0.69 to 2.13)	300 (1 RCT)	⨁⨁◯◯ LOW [c]	
Volume of blood loss	The mean volume of blood loss ranged from 441.7 to 605.1 mL	The mean volume of blood loss was **146.77 mL lower** (168.10 lower to 125.43 lower)	-	601 (3 RCTs)	⨁⨁⨁◯ MODERATE [d]	
Incidence of blood transfusion	40 per 1.000	**20 per 1.000** (5 to 78)	**RR 0.50** (0.13 to 1.95)	301 (2 RCTs)	⨁⨁◯◯ LOW [a]	
Incidence of serious morbidity -hysterectomy	0 per 1.000	**0 per 1.000** (0 to 0)	**RR 3.00** (0.12 to 72.77)	301 (2 RCTs)	⨁⨁◯◯ LOW [e]	

*__The risk in the intervention group__ (and its 95% confidence interval) is based on the assumed risk in the comparison group and the **relative effect** of the intervention (and its 95% CI).
CI: Confidence interval; **RR:** Risk ratio; **MD:** Mean difference

GRADE Working Group grades of evidence
High certainty: We are very confident that the true effect lies close to that of the estimate of the effect
Moderate certainty: We are moderately confident in the effect estimate: The true effect is likely to be close to the estimate of the effect, but there is a possibility that it is substantially different
Low certainty: Our confidence in the effect estimate is limited: The true effect may be substantially different from the estimate of the effect
Very low certainty: We have very little confidence in the effect estimate: The true effect is likely to be substantially different from the estimate of effect

Explanations
a. Evidence certainty downgraded -2 due to very serious imprecision (very small number of events, and wide 95% CI crossing the line of no effect).
b. Evidence certainty downgraded -1 due to serious imprecision (small sample size and wide 95% CI compatible with no effect or an important benefit favoring the group that received oxytocin before fetal delivery).
c. Evidence certainty downgraded -1 due to risk of bias (lack of blinding of participants, subjective outcome) and -1 due to serious imprecision (small sample size and wide 95% CI crossing the line of no effect)
d. Evidence certainty downgraded -1 due to serious imprecision (small sample size).
e. Evidence certainty downgraded -2 due to very serious imprecision (lack of events)
