# Supplementary material for: Timing of oxytocin administration to prevent post-partum hemorrhage in women delivered by cesarean section: A systematic review and metanalysis
Source: PLoS One. 2021 Jun 3;16(6):e0252491. doi: 10.1371/journal.pone.0252491 (PMC8174699; doi:10.1371/journal.pone.0252491)
Supplement: S8 Table — Summary of findings table and GRADE for comparison 2: Prophylactic oxytocin administered before versus after placental separation. (PDF) [file pone.0252491.s009.pdf]

S8 Table. Summary of findings table and GRADE: prophylactic oxytocin administered after fetal delivery, Before versus After Placental separation at cesarean section

Summary of findings

Patient or population: women giving birth by cesarean section
Setting: hospital
Intervention: oxytocin given Before Placental Separation
Comparison: oxytocin given After Placental Separation

Outcomes	Anticipated absolute effects* (95% CI)		Relative effect (95% CI)	№ of participants (studies)	Certainty of the evidence (GRADE)	Comments
	**Risk with oxytocin given After Placental Separation**	**Risk with oxytocin given Before Placental Separation**				
Incidence of PPH ≥ 1000 mL	0 per 1.000	**0 per 1.000** (0 to 0)	not estimable	100 (1 RCT)	⊕◯◯◯ VERY LOW [a]	One study [Mangla 2012] reported that no patients in both groups (0/50 versus 0/50) had blood loss ≥ 1000 mL
Need for additional uterotonic	0 per 1.000	**0 per 1.000** (0 to 0)	not estimable	100 (1 RCT)	⊕◯◯◯ VERY LOW [a]	One study [Mangla 2012] reported that no patients in both groups (0/50 versus 0/50) needed additional uterotonics
Adverse effects of oxytocin - nausea/vomiting	20 per 1.000	**20 per 1.000** (1 to 311)	**RR 1.00** (0.06 to 15.55)	100 (1 RCT)	⊕◯◯◯ VERY LOW [b]	

***The risk in the intervention group** (and its 95% confidence interval) is based on the assumed risk in the comparison group and the **relative effect** of the intervention (and its 95% CI).

CI: Confidence interval; **RR:** Risk ratio

GRADE Working Group grades of evidence
High certainty: We are very confident that the true effect lies close to that of the estimate of the effect
Moderate certainty: We are moderately confident in the effect estimate: The true effect is likely to be close to the estimate of the effect, but there is a possibility that it is substantially different
Low certainty: Our confidence in the effect estimate is limited: The true effect may be substantially different from the estimate of the effect
Very low certainty: We have very little confidence in the effect estimate: The true effect is likely to be substantially different from the estimate of effect

Explanations
a. Evidence certainty downgraded -1 due to risk of bias (unclear risk for selection bias and selective reporting) and -2 due to very serious imprecision (lack of events)
b. Evidence certainty downgraded -1 due to risk of bias (lack of blinding of participants-subjective outcome, unclear risk for selection bias and selective reporting) and -2 due to very serious imprecision (very small number of events, and wide 95% CI crossing the line of no effect).
