# Supplementary material for: Timing of oxytocin administration to prevent post-partum hemorrhage in women delivered by cesarean section: A systematic review and metanalysis
Source: PLoS One. 2021 Jun 3;16(6):e0252491. doi: 10.1371/journal.pone.0252491 (PMC8174699; doi:10.1371/journal.pone.0252491)
Supplement: S1 Text — (PDF) [file pone.0252491.s010.pdf]

Prophylactic oxytocin to prevent postpartum hemorrhage at cesarean section

Maria Regina Torloni, Monica Siaulys, Rachel Riera, Ana Pilar Betran, Mariana Widmer

To enable PROSPERO to focus on COVID-19 registrations during the 2020 pandemic, this registration record was automatically published exactly as submitted. The PROSPERO team has not checked eligibility.

Citation

Maria Regina Torloni, Monica Siaulys, Rachel Riera, Ana Pilar Betran, Mariana Widmer. Prophylactic oxytocin to prevent postpartum hemorrhage at cesarean section. PROSPERO 2020 CRD42020186797 Available from:
https://www.crd.york.ac.uk/prospero/display_record.php?ID=CRD42020186797

Review question

In women submitted to cesarean section, what is the effectiveness and safety of prophylactic oxytocin administered in different periods, regimens, routes, and doses?

Searches

We will develop a search strategy combining key words related to oxytocin and cesarean delivery that will be adapted and run in several databases such as PubMed, Embase, CENTRAL and CINAHL. There will be no date or language limitations.

We will also create and run a search for ongoing studies in trial registry platforms.

The electronic search will be complemented by screening the reference lists of all included studies and other systematic reviews on prevention of postpartum hemorrhage (PPH).

Types of study to be included

All randomized controlled trials of effectiveness or side-effects of oxytocin versus oxytocin to prevent PPH in women submitted to CS will be included.

Condition or domain being studied

Prevention of postpartum hemorrhage. Cesarean section. Oxytocin

Participants/population

Inclusion criteria:

We will include studies that enrolled women of any age, race, socioeconomic level, nationality, with or without any health problems, at high or low risk for PPH, who delivered by cesarean section (CS) for any indication, with any number of fetuses, and at any gestational age after 26 weeks. We will include trials that enrolled women who had a primary or a repeat CS, a prelabour (planned or emergency) CS, or an intrapartum CS. We will include studies where participants received oxytocin for labor induction or augmentation before the CS. Studies that tested oxytocin to prevent PPH in participants having vaginal and cesarean deliveries will be included if the authors present data separately for the women having a CS.

Exclusion criteria:

We will exclude studies that assessed oxytocin in combination with another uterotonic drug, or that compared oxytocin versus placebo or other uterotonic drugs to prevent PPH.

Intervention(s), exposure(s)

We will compare different routes, dosages, regimen, or periods of administration of oxytocin to prevent PPH in women submitted to CS. We will include trials in which non-pharmacologic co-interventions (e.g. uterine massage) were included as a randomized intervention in all arms of the trial.

Multi-arm trials that compared oxytocin versus other uterotonic drugs, but that also compared different routes, dosages, regimens or periods of administration of oxytocin alone will be included.

Comparator(s)/control

Any route, dosage, regimens or periods of administration of oxytocin to prevent PPH at CS that are different from the intervention group.

Main outcome(s)

1. Postpartum hemorrhage of at least 1000 mL assessed by any method
2. Need for additional uterotonics

3. Any side effects

* Measures of effect

Incidence, Relative risk. We will use other effect measures depending on the data reported in the trials.

Additional outcome(s)

1. Volume of blood loss (mL) measured by any method

2. Blood transfusion after placental delivery

3. Shock, as defined by trialists

4. Maternal mortality or severe maternal morbidity related to PPH (organ failure, coma, hysterectomy, or as defined by the study authors)

5. Maternal transfer to a higher level of care

6. Maternal satisfaction (or sense of wellbeing) as defined by trialists

7. Breastfeeding at discharge

* Measures of effect

Incidence, Relative risk. We will use other effect measures depending on the data reported in the trials.

Data extraction (selection and coding)

The references retrieved from each database will be uploaded into a reference management software and duplicates will be excluded. Two review authors will independently screen titles and abstracts to select potentially relevant records. Studies selected at first screening will be read by two independent authors who will include those that fulfill the selection criteria. Reasons for exclusion will be listed. Disagreements in these two phases will be solved by a third review author.

Two authors will independently conduct data extraction. Disagreements will be solved by a third review author. We will contact study authors by email to obtain missing information and study details, when necessary.

We will use a form created for this review to extract data from each included study. The data will be entered in the Review Manager software and checked for accuracy. The following information will be collected from each study: country, year, trial registration, design details, number of participants, inclusion and exclusion criteria, main participant characteristics, type of CS, anesthesia, previous exposure to oxytocin, interventions compared and outcomes reported.

Risk of bias (quality) assessment

The risk of bias of each included study will be assessed independently by two review authors. Disagreements will be solved by a third reviewer. Full justifications for the assessments will be included in the report.

We will use the Cochrane Risk of Bias (RoB) tool to assess the quality of included trials. The following domains will be assessed and graded as being at high, low, or unclear risk of bias: 1) Random sequence generation, 2) Allocation concealment, 3) Blinding of participants and personnel, 4) Blinding of outcome assessment, 5) Incomplete outcome data, 6) Selective reporting (checking for reporting bias), 7) Other bias.

Strategy for data synthesis

We will pool data of similar trials in metanalyses using the random effect model using the Review Manager software (version 5.3). For dichotomous outcomes, we will present results as summary risk ratio (RR) with 95% confidence intervals (CIs). For continuous outcomes, we will pool the results of studies that measured the outcome in the same way and calculate mean differences (MD). In metanalyses of studies that measured the same continuous outcomes using different methods, we will use the standardized mean difference (SMD).

When metanalysis is not possible, we will present the results descriptively.

We will conduct separate comparisons for prelabor and intrapartum CS.

We plan to conduct the following comparisons, where data is available:

• different moments of administration.

• different routes of administration

• different regimens

• different doses

Analysis of subgroups or subsets

We plan to conduct the following subgroup analyses for the primary outcomes, if data are available: according to pregnancy characteristics (parity, onset of labor), according to previous use of oxytocin for induction/augmentation, and according to baseline risk for PPH. We will assess subgroup differences by interaction tests available in RevMan 5 (RevMan 2014).

We plan to conduct the following sensitivity analyses for the main outcomes, if data are available: restricted only to high-quality studies, and restricted only to studies that assessed blood loss objectively.

Contact details for further information

Maria Regina Torloni
torlonimr@gmail.com

Organisational affiliation of the review

Hospital e Maternidade Santa Joana

Review team members and their organisational affiliations

Dr Maria Regina Torloni. Hospital e Maternidade Santa Joana
Dr Monica Siaulys. Hospital e Maternidade Santa Joana
Dr Rachel Riera. Universidade Federal de Sao Paulo
Dr Ana Pilar Betran. World Health Organization
Dr Mariana Widmer. World Health Organization

Collaborators

Dr Ligia Mathias. Hospital e Maternidade Santa Joana
Dr Guilherme Haelvoet. Hospital e Maternidade Santa Joana
Dr Ana Luiza Martimbianco. Universidade Federal de Sao Paulo
Carolina Cruz Latorraca. Universidade Federal de Sao Paulo
Dr Rafael Leite Pacheco. Universidade Federal de Sao Paulo

Type and method of review
Intervention, Meta-analysis, Systematic review

Anticipated or actual start date
06 April 2020

Anticipated completion date
31 July 2020

Funding sources/sponsors
Department of Sexual and Reproductive Health and Research, World Health Organization

Conflicts of interest

Language
English

Country
Brazil, Switzerland

Stage of review
Review Ongoing

Subject index terms status
Subject indexing assigned by CRD

Subject index terms
MeSH headings have not been applied to this record

Date of registration in PROSPERO
05 July 2020

Date of first submission
17 May 2020

Stage of review at time of this submission

Stage	Started	Completed
Preliminary searches	Yes	No
Piloting of the study selection process	Yes	No
Formal screening of search results against eligibility criteria	No	No
Data extraction	No	No
Risk of bias (quality) assessment	No	No
Data analysis	No	No

The record owner confirms that the information they have supplied for this submission is accurate and complete and they understand that deliberate provision of inaccurate information or omission of data may be construed as scientific misconduct.

The record owner confirms that they will update the status of the review when it is completed and will add publication details in due course.

Versions

05 July 2020

PROSPERO
This information has been provided by the named contact for this review. CRD has accepted this information in good faith and registered the review in PROSPERO. The registrant confirms that the information supplied for this submission is accurate and complete. CRD bears no responsibility or liability for the content of this registration record, any associated files or external websites.
